# Strengthening adolescent well-being—controlled feasibility from two Swedish upper secondary schools

**DOI:** 10.1186/s12889-026-27307-2

**Published:** 2026-04-15

**Authors:** Lisbeth Kristiansen, Niklas Boman, Niclas Olofsson

**Affiliations:** 1 Department of Health Sciences, Faculty of Human Sciences, Mid Sweden University, Holmgatan 10, 85170 Sundsvall, Sweden; 2 Department of Children and Education Administration, Timrå Municipality, Sweden; 3 Department of Communication, Quality Management and Information Systems, Mid Sweden University, Sundsvall, Sweden

**Keywords:** Health promoting, students' well-being, school based universal intervention

## Abstract

**Background:**

Positive mental health refers to a state of well-being in which children and young people realize their own abilities, learn to cope with the everyday stresses of life, develop a positive sense of identity, learn to manage thoughts and emotions, build social relationships, and acquire education that fosters active citizenship. There is a need for a stronger evidence base regarding mental health promotion to address mental health issues in the young generation. To further help bridge this gap, this study compared and evaluated a universal health-promoting intervention facilitated by Student Health Care Teams (SHCTs) for Grade 12 students at two schools, and assessed its effects on students' self-reported well-being, resilience, strengths and difficulties, and mental health. Furthermore, the study aimed to qualitatively evaluate the intervention from students’ and facilitators’ perspectives.

**Method:**

This non-randomized feasibility study used an explanatory sequential mixed-methods design (MRC), which exploratory compared pre- and post-outcomes between boys and girls, and qualitative, deductive content analysis to make sense of the data. Comparisons between the experimental (*n* = 44) and control groups (*n* = 41) were made in terms of several self-reported measurements: the WHO-5, a short and generic global rating scale, which measures psychological well-being as the primary outcome; the Resilience Scale (RS); strength, which is measured by the Strengths and Difficulties Questionnaire (SDQ); and mental health, which is measured by the Hospital Anxiety and Depression Scale (HADS). Furthermore, there were nine focus group interviews (42 students in 5 groups; 10 SHCT members in 4 groups).

**Results:**

Controlling for pre-intervention values (WHO-5, RS, HADsA, HADsD, and SDQ), the intervention increased the OR scores of the girls above post-intervention values on the WHO-5 (OR = 9.0; 1.4–56). The intervention did not improve the OR of higher scores in boys during follow-up (OR = 0.1; 0.09–1.4). Students and SHCTs generally found the intervention feasible.

**Conclusion:**

Girls appeared to benefit more from the intervention than boys, suggesting a need for gender-specific approaches. Despite time demands, school health teams valued the model for supporting interprofessional, school-based health promotion. However, larger studies are needed to confirm these findings.

## Introduction

This paper describes the second feasibility study for the Strengthening Adolescents Wellbeing (SAW), an universal school-based health promotion intervention that student health care teams facilitate at classroom level without teachers. SAW has been developed according to outreach and recommendations of the European Commission [1] as means to improve mental health among young people. In summary, the former results were promising, leading us to continue expanding the intervention [2, 3].

### Background

In the twenty-first century, children and young people face several health-related challenges. Both physical factors, such as a high intake of total fat, free sugars, and salt, along with a lack of physical activity, contribute to increasing children’s obesity at alarming rates, as do mental health challenges in adolescents [4]. The prevalence of mental health illness and disease among children and young adults is increasing, which is still a significant area of concern [5]. Mental disorders often occur early in life, posing a significant global health challenge and hindering young individuals from completing age-appropriate activities during crucial developmental phases. Common disorders include depression, anxiety, disruptive behaviour disorders, attention hyperactivity disorders, and substance use disorders, all of which have notable impacts, particularly on children and adolescents (see, for instance, [6]).

In a cross-sectional, cross-national study within the Health Behaviour in School-aged Children (HBSC) from 2002–2018, Cosma et al. [7] did not find evidence of substantial declines in mental well-being among adolescents. Still, girls presented a much greater risk of both psychosomatic health complaints and low life satisfaction than boys did. Based on the HBSC 2021/22 survey, Inchley et al. [8]. Assessing life satisfaction and comparing adolescents across five Nordic countries reveals diverse trends, with Norway and Sweden showing different levels of life satisfaction Due et al. [9]. Socioeconomic disparities in mental health and well-being of adolescents persisted across Nordic countries during the 2000 s [10]. In Sweden, economic inequality has risen more than in other Nordic countries. The rise in income inequality might be linked to higher levels of self-reported health complaints (SHCs) and lower life satisfaction in Sweden compared with its Nordic sister countries [10].

In a systematic review of universal programs aimed at promoting children's and adolescents’ mental well-being, with a focus on interventions delivered in schools or to families, SBU [11] investigated the effects of the programs, participant experiences, and health, economic, and ethical aspects. SBU [11] concluded, in brief,that social and emotional learning (SEL) programs have moderate effects on social skills, resilience, and emotional competence; mindfulness and yoga may also support well-being. This was especially true for girls; students and teachers generally responded positively, although ethical concerns were noted; and long-term effects and cost-effectiveness remained unclear due to limited data [11]. In another recent Swedish study by Hermann et al. [12], a dual-factor model was developed to better understand different patterns in four identified subgroups of adolescents (*vulnerable, complete mental health, troubled,* and *symptomatic but content*) in relation to mental health status. The model helps identify *vulnerable youth* (about 50% of the 1,833 respondents) who might otherwise be overlooked, highlighting the need for interventions that promote well-being and address resilience, stress, and gender norms [12].

However, there is still a need for a broader and stronger evidence base regarding mental health promotion to address mental health issues fully in the younger generation [13]. The European Commission [14] states that schools across Europe should prioritize and actively promote the mental health and well-being of schoolchildren within safe and inclusive contexts. A holistic approach to education in line with the social, emotional, and physical needs of children and young people, and a focus on well-being and mental health as key learning goals. In a more holistic view of individual development, the primary commitment of school systems—alongside students’ academic achievements—should be the improvement of young people’s physical, mental, and social well-being [15]. Schools may represent the optimal setting for educational health-related interventions, as educators can positively influence students’ lifelong learning and work to reduce health inequalities among young people [16].

A comprehensive school commitment to students’ global well-being is expected to positively impact children’s behaviours. For that reason, we question why educational institutions are not widely and systematically engaged on a proper path—according to the WHO—toward becoming “health-promoting schools” able to prevent students’ harmful behaviours [15].

Furthermore, from a professional school and student health care perspective, interventions are needed, as school health care teams (SHCTs) must prioritize promoting health through group-level approaches, according to the Swedish Act on School [17]. These teams consist of registered school nurses, social workers, and special education teachers who offer support talks and special support in the classroom. For more contextual information about Swedish student health care teams, please read, for example, the development of school children’s health [18].

In this study, we focus on the World Health Organization’s [5] definition of positive mental health as a state of well-being where children and young people realize their own abilities, learn to cope with everyday stresses of life, develop a positive sense of identity and the ability to manage thoughts and emotions, build social relationships, and acquire an education that fosters active citizenship.

**Fig. 1:**
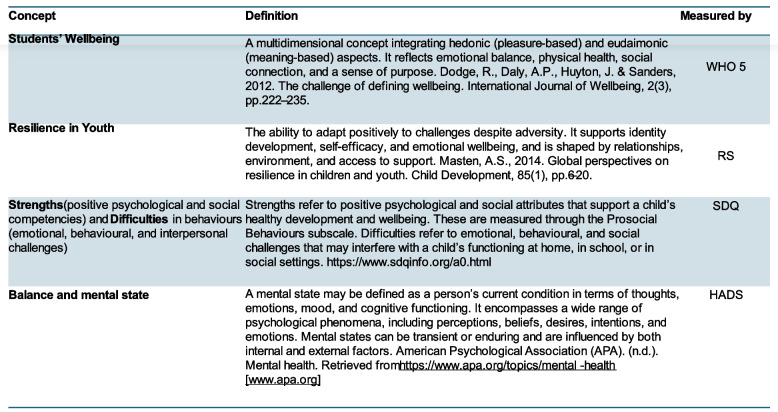
Shows how the key concepts are measured in this study

The SAW intervention aims to promote students’ well-being and mental health by fostering, supporting, and elaborating positive mental states. In Fig. 1 below, we provided a definition of the key concepts: Students’ well-being, Resilience Youth, Strengths and difficulties, and Balance and mental state, and how we have operationalised them. The intervention is designed to strengthen protective factors, including self-esteem, self-efficacy, and social inclusion. This is achieved through interaction with SHCTs in a safe and supportive environment, where the intervention components are provided. The SAW intervention components are knowledge dissemination (essential aspects of health—social, physical, psychological, and sexual, exposure to violence, healthy lifestyle habits, healthy eating, gender and equality), stress management, reflective exercises, and creative activities (See [2, 3]). SAW begins with the researchers’ training of SHCT members in the intervention's structure and content. Teachers divide their classes into small groups (8–12 students) that meet with the SHCT during 1.5-h sessions for 8 weeks. Each session follows the same structure; lighting of a candle, reading a mindful relaxation text, having a lecture on the topic of the session, sharing experiences, reflecting on the subject, creative and playful activity, eating fruit, short discussion, presentation of subsequent sessions’ topic, and finally, blowing out the candle (See [2, 3]). The specific composition of content components and the specific context in which SHCTs facilitate the intervention at the classroom level are, to the best of our knowledge, unique.

The purpose of the current study was to compare and evaluate the SAW intervention, facilitated by the SHCTs, for upper secondary school students in Grade 12, and to examine its effects on students' self-reported well-being, mental health, resilience, and perceived stress. Furthermore, to qualitatively evaluate the feasibility of the intervention from the student and facilitator perspectives, to explore Saw’s acceptability among students and SHCTs, and whether the implementation of SAW can give preliminary signals of effectiveness, we posed the following research questions:

- RQ 1: Can participation in the SAW intervention, facilitated by School Health Care Teams (SHCTs), lead to significant improvements in self-reported wellbeing? (primary outcome, measured by WHO-5).
- RQ2: Can participation in the SAW intervention improve resilience, strengths, balance, and mental state, and can it reduce difficulties among Grade 12 upper secondary school students? (Secondary outcomes measured by RS, SDQ, and HADS are explanatory).
- RQ3: Do students and SHCTs perceive the intervention as feasible?

## Method

We used a non-randomized feasibility study with an explanatory sequential mixed method and comparative design [19], which is in accordance with the recommendations from the United Kingdom Medical Research Council on the development and evaluation of complex interventions in an early phase of assessing feasibility before a complete review [20]. We are inspired by the Nexus Holistic Wellbeing Framework, which includes a systems-based, interdisciplinary model for schools that integrates emotional, social, physical, and academic wellbeing [21]. This model promotes strengths-based, inclusive practices and calls for whole-school policies that support diverse student needs through collaboration and systemic thinking [21]. We followed the CONSORT 2010 statement: extension to randomised pilot and feasibility trials guideline [22]. 

### Settings and participants

As this is an upscaling of a previous pilot study, conducted in conjunction with the local public organizer of upper secondary school, this round of SAW included more students and staff than the first round [2]. The schools identified a need to improve health promotion work with their students. The schools in our setting had approximately 2200 students and were situated in the city centre, in the public school system under the authority of the municipality's main school director. A suitable scope for the intervention was developed by the researchers and the schools, accounting for which classes could be allocated to the intervention and control groups and ensuring that SHCTs could participate. The main school director approved the research and formally decided to include the students in the intervention or control groups. At the same time, the main school director randomly selected which school would serve as the control group and which would serve as the intervention group. This meant that at the first level, schools were randomly selected; at the second level, school classes were conveniently sampled; and at the third level, all students in the sampled classes were invited to participate in the intervention. This procedure disqualified the design from being fully randomised; it was more akin to a cluster-randomised design. In future studies, we plan to use a cluster-randomised design. The main school director also decided that the intervention was a mandatory part of the ordinary curriculum.

For participants, admission scores differed between the intervention and control groups; the average admission score in the intervention group was 190, and in the control group, 254. The maximum score in the Swedish system was 320 points (340 points with extra credit), indicating the highest grade across all topics, whereas the lowest passing grade in all subjects yields an admission score of 170 points. The students attended different study programs, each with distinct admission scores. Although the students in this study faced similar academic challenges, the intervention and control groups were not matched. The intervention and control schools may represent children from across the socioeconomic spectrum, which was not evaluated. The students generally started the first year of Swedish upper secondary school, eleventh grade, at 16 years of age. In general, 15% of adolescents who do not continue to secondary school after primary school do so because of their admission score. Rather, they chose to start working, become trainees, or become sick, which hindered them from attending secondary school at that time.

### Sample recruitment

After obtaining permission from the main school director, the research team contacted the two public upper secondary school principals and the heads of the participating students. To be eligible for the intervention, students had to meet the inclusion criteria, i.e., be able to speak and understand Swedish at the level required in secondary school. There is a lack of a universally accepted minimum clinically significant difference using WHO-5 (see, for instance, [23]). Earlier research, however, has indicated that a change of 10 points is relevant (see, for example, [24]). The researchers estimated that they needed 98 students to detect a 10% change in the WHO-5 (the primary outcome) with α = 0.95 and β = 0.20. During school hours, the researchers provided students and SHCTs with verbal information about the study's details. That participation was voluntary, whereas participation in the sessions was integrated into the ordinary curriculum. All the students provided written consent to participate. Overall, 91 students were recruited from the second year of upper secondary school to the study and were convenience sampled into an intervention group (*n* = 47) and a control group (*n* = 44). The students were between 17 and 20 years old.

### Description of the intervention

Before implementing the intervention in classrooms, the researchers led workshops and practical exercises for the SHCT personnel over three days, during which the SHCTs were trained in the SAW format and sessions and instructed on how to lead and deliver the SAW. After the training, the SHCTs delivered and facilitated the intervention at the classroom level to students with whom they were familiar and comfortable [2]. The control group received “student health care as usual”. The core components of the SAW intervention were knowledge dissemination, stress management, reflective exercises, and creative activities. While the adaptable components were lighting a candle, reading a mindful relaxation text, eating fruit, presenting the topic of the subsequent session, and blowing out the candle. Please see Table 1.

### Data collection

#### Data collection of the quantitative data

The questionnaires were completed individually by the students before and one month after the intervention was finalized. Two to three researchers were present in the classroom and read the questionnaire items aloud, making clarifications if needed.

### Instrumentations

WHO-5 [25], a short and generic global rating scale, to measure self-reported psychological well-being, as well as the primary outcome. The WHO-5 adequately measures well-being in research, serves as a screening instrument for depression, and is an outcome measure in clinical trials [26].

The Resilience Scale (RS) [27] was used to measure students’ ability to cope with everyday challenges. The RS comprises 25 items and employs a seven-point Likert-type scale ranging from 'strongly disagree' to 'strongly agree'. It encompassed dimensions such as health-promoting activities, forgiveness, stress, and anxiety, for instance, statements such as “I can usually find something to laugh about”. In terms of reliability and validity, the RS has proven to be an effective tool for assessing resilience and has been successfully employed across diverse study populations [27]. An established Swedish version of RS was used [28].

The Strengths and Difficulties Questionnaire (SDQ), which is often used to gain insight into the mental health of children and adolescents [29, 30], was used as a measure of mental health. The SDQ is a 25-item instrument that concerns attributes, some positive and some negative, of the respondent, covering five areas of psychological dimensions [31]. It reflects the following problem areas: emotional symptoms, conduct problems, hyperactivity-inattention; peer problems or strengths; mental strengths; and prosocial behaviours [32]. It was reached on a website that provided unrestricted use.

To reflect students’ balance and mental state, the Hospital Anxiety and Depression Scale (HADS) was used. With 14 questions across two subscales: anxiety (HADS-A) and depression (HADS-D) [33] should be reference number 33. The sensitivity and specificity for both the HADS-A and HADS-D of approximately 0.80 were very similar to the sensitivity and specificity achieved by the General Health Questionnaire (GHQ) [34].

### Data collection of the qualitative data

To evaluate the intervention, nine focus group interviews were conducted by one or two researchers [35]. Altogether, 42 students participated in five groups, with an average of 8 students per group, grouped by the SAW they had joined. We used a guideline with questions to explore how the student had experienced their participation in the intervention, as well as the intervention's content and format. We, for example, asked:” *How did you experience being a part of SAW?”, “What are your thoughts on SAW content and form*?”, “*What, in your opinion, was good, and not so good about it?*”. The interviews were recorded and lasted as long as the students needed to elaborate on the questions.

To be included in the SHCT focus groups, the staff had to have been engaged during all eight weeks of intervention. We developed a semi-structured question guide, where we, for instance, asked:” What *is your overall impression of the SAW intervention?*” “*How did you experience facilitating the SAW?” “What are your thoughts on SAW content and form*?” There were between two and four SHCT members in four focus group interviews (10 unique individuals in total), and the discussions lasted between 45 and 56 min.

### Data analysis

#### Data analysis of the questionnaires

Descriptive statistics, including the mean, standard deviation, and range, were used to summarize results across the different questionnaires (*n* = 91). Paired Student t-tests were used to compare the before and after mean values in the different groups. Owing to a tendency of regressions towards the mean [36], and because we wanted to isolate the outlines, we focused further analyses on the intermediate group according to the following definition: the intermediate group was defined as being above the 12.5th percentile (*n* = 7 school students, *n* = 2 from the experimental group and *n* = 5 from the control group), the lower 87.5th percentile (*n* = 10 school students, *n* = 6 from the experimental group and *n* = 4 from the control group), and the WHO 5th measurement pretest time (baseline) (time 1). This accounted for 75% of the total sample and included 71 students: 36 in the experimental group (11 boys, 25 girls) and 35 in the control group (17 boys, 18 girls). Missing values can arise from information loss, as well as from dropouts and non-responses among study participants. The presence of missing values reduces the sample size and compromises the reliability of the study results. An exploratory and post hoc robust logistic regression analysis [37] was performed to determine whether the intervention affected the primary outcome, the WHO-5. The baseline values of the WHO-5, RS, SDQ, HADS-A, and HADS-D were controlled for. As this was a feasibility study, the analyses were aligned to keep them simple and robust. In a future study using a different design might be considered.

#### Data analysis of the interviews

A deductive design was used when performing the content analysis of the transcribed group interviews [38]. All the transcriptions were initially read to get a sense of the whole before the interviews were coded. All applicable interview texts were sorted to systematically organize and make sense of the data, using a categorization matrix. The matrix is based on a previously conducted qualitative content analysis that identified essential factors, such as “experienced values and overall opinions”, “SAW’s educational content and experimental content”, and “SAW’s format” [3].

### Ethics approval and consent to participate

The study was assessed and approved by the Swedish Ethical Review Authority, the regional committee in Umea (No. 2016–363-32 M, and No. 2018–321–322019–06333). The principles of research and researcher ethics outlined in the Declaration of Helsinki were followed at every step of the research process. Each student and student health care team member was informed by the researcher twice, with a thorough briefing beforehand and a guarantee of confidentiality. All participants provided written consent, indicating that informed consent was obtained. Furthermore, they were informed that their participation was voluntary and that they could withdraw from the research at any time without consequences.

### Consent for publication

Each student and student health care team member provided consent for publication in a scientific journal (Table 1).

**Table 1:** Content of the teaching dimensions in the intervention

*Dimensions of well-being*	*Examples of the content of education program sessions*
Mental health and wellbeing	How to achieve mental health and wellbeing? Health-promoting and supportive factors? How to seek support if common mental disorders such as depression and anxiety, NPF arise?
Physical health	Healthy lifestyle, physical activity, sleep
Social health	Social capital, social support, and structural factors
Gender, equity, and involvement in school	Swedish legislation, power structures, intersectionality, and the school as an arena of fostering democracy
Sexual health	Was it good relations, sexual behavior, and pornography?
Digital and IRL Bullying and harassment	Bullying, harassment, sexual harassment, and the school environment
Healthy eating	What does healthy eating mean?
Stress	Stress reactions, coping, and recovery

## Results

To develop a complex intervention, several steps are needed. In line with the explanatory sequential mixed-methods approach, quantitative results are presented first, followed by a qualitative report of the findings. A statistically significant difference between the experimental and control groups was found, as well as a difference between girls and boys within these groups.

There was a general improvement across all measurements in the entire study population. Comparisons of all students at baseline (time 1) and postintervention (time 2), and between the intermediate group at baseline and postintervention, were reported as mean scores and standard deviations. These results persisted in the intermediate group (Table 2).

**Table 2:** Measurements at baseline and post-intervention: mean (standard deviation)

	**Total** ***n* = 91** **Baseline**	**Post-intervention**	**Intermediate group 1** ***n* = 71** **Baseline**	**Post-intervention**
Well-being index (WHO-5)	55,0 (22,3)	62,1* (21,0)	54,5 (16,6)	61,8*(22,2)
Resilience scale (RS)	103 (18,3)	106 ns (11,6)	103 (15,1)	105 ns (16,6)
Strengths and Difficulties Questionnaire (SDQ)				
Pro social	9,16 (3,79)	8,79 ns (1,44)	8,86 (1,41)	8,8 ns (1,32)
Hyperactivity	4,47 (2,19)	3,72 ns (2,16)	4,47 (2,01)	3,69 ns (2,17)
Emotional problems	3,70 (2,47)	3,57 ns (2,44)	3,67 (2,31)	3,46 ns (2,49)
behaviour problems	1,95 (1,61)	1,83 ns (1,48)	1,77 (1,34)	1,82 ns (1,58)
Peer problem	2,48 (1,72)	2,37 ns (1,72)	2,45 (1,58)	2,33 ns (1,81)
Total	12,6 (5,46)	11,5 ns (5,55)	12,4 (4,37)	11,3 ns (5,86)
The Hospital Anxiety and Depression Scale				
HADS- A	8,62 (4,33)	7,49 ns (4,24)	8,44 (3,64)	7,54 ns (4,48)
HADS-D	5,73 (3,78)	4,36* (3,32)	5,37 (3,18)	4,38 ns (3,39)

1. In-between the 12,5:th percentile and the 87,5:th percentile

2. ns = not significant, * = < = 0.05, ** = < = 0.01, *** < = 0.001

The WHO-5 scores of both the whole study group and the intermediate groups significantly improved between the interventions. The RS scores showed a positive change, but it was not statistically significant. HADS-A and HADS-D both differed between baseline and postintervention in both the total and intermediate groups. They were all lower at follow-up in both groups and both scores. Only HADS-D in the total group was statistically significantly different between pre- and postintervention. The results from the SDQ sub-scales showed no significant differences. All in all, the scores remained unchanged or slightly lower from baseline to time postintervention.

In total, this could be summarized as a positive change in the primary outcome, WHO-5, and in the secondary outcomes, RS, HADS-A, and HADS-D. While the secondary outcome, SDQ score, did not change.

A comparison between girls in intervention groups at time 1 and time 2 and girls in the control group is presented in Table 3. The girls in the intervention group reported higher WHO-5 scores at follow-up, whereas those in the control group reported slightly lower scores. The higher WHO-5 score in the intervention group and the lower score in the control group were statistically significant. The same tendencies were found in both the SDQ's emotional problems dimension and the HADS-A. The intervention group showed slightly lower scores in the two problem dimensions, and the control group showed higher scores in the respective dimensions. Here, the differences between baseline and postintervention were also statistically significant.

**Table 3:** Comparison between girls in the intervention and control groups at baseline (time 1) and post-intervention (time 2), reflected in mean scores and standard deviations

	**Intervention group** **Baseline**	**Post-intervention**	**Control group** **Baseline**	**Post-intervention**
Well-being index (WHO-5)	50,4 (14,3)	62,9 * (20,4)	55,8 (19)	48,5* (19,6)
Resilience scale (RS)	98,5 (15,2)	102 ns (16,4)	107 (13,4)	105 ns (18,4)
Strengths and Difficulties Questionnaire (SDQ)				
Pro social	8,60 (1,50)	8,73 ns (1,28)	8,61 (1,65)	9,47 ns (0,72)
Hyperactivity	4,60 (1,87)	4,05 ns (2,42)	4,39 (1,94)	3,29 ns (1,61)
Emotional problems	4,24 (2,06)	3,0* (2,35)	3,83 (2,79)	4,18* (2,48)
Behaviour problems	2,12 (1,56)	1,95 ns (1,53)	1,50 (1,38)	1,18 ns (0,73)
Peer problem	2,60 (1,38)	2,55 ns (2,06)	2,27 (1,77)	2,18 ns (1,38)
Total	13,6 (3,88)	11,5 ns (6,33)	12,0 (4,77)	10,8 ns (3,66)
The Hospital Anxiety and Depression Scale				
HADS- A	8,68 (3,00)	6,0* (2,92)	9,78 (4,67)	11,3* (5,77)
HADS-D	5,48 (2,54)	4,57 ns (3,25)	5,88 (4,25)	4,88 (3,74)

1. ns = not significant, * = < = 0.05, ** = < = 0.01, *** < = 0.001

When boys in the intervention and control groups were compared, the results, with some exceptions, were the opposite of those of the girls (Table 4). There were some unexpected differences in the boy’s intervention group. For instance, in WHO-5, a slightly lower score at time 2 than at baseline, and a somewhat higher score on the SDQ-dimension emotional problems and the HADS-D. However, the prosocial dimension of the SDQ was higher at postintervention than at baseline.

**Table 4:** Comparison of boys between the intervention and control groups at baseline and post-intervention, presented as means and standard deviations

	**Intervention group** **Baseline**	**Post-intervention**	**Control group** **Baseline**	**Post-intervention**
Well-being index (WHO-5)	58,2 (18,4)	56,6* (26)	56,7 (16,3)	76 ns (17,6)
Resilience scale (RS)	108 (17,6)	96,3 ns (13,3)	101 (14,4)	113 ns (14,2)
Strengths and Difficulties Questionnaire (SDQ)				
Pro social	8,90 (1,28)	9,43* (0,79)	9,47 (0,87)	8,29 ns (1,72)
Hyperactivity	4,50 (2,06)	4,57 ns (2,23)	4,35 (2,37)	3,30* (2,31)
Emotional problems	2,20 (1,68)	4,86* (2,34)	3,53 (2,21)	2,76* (2,54)
Behaviour problems	2,10 (0,99)	3,14 ns (2,61)	1,35 (0,99)	1,76 ns (1,52)
Peer problem	3,30 (1,82)	3,0 ns (2,52)	1,94 (1,39)	1,94 ns (1,56)
Total	12,1 (4,72)	15,6 ns (8,04)	11,2 (4,37)	9,76 ns (5,67)
The Hospital Anxiety and Depression Scale				
HADS- A	6,00 (3,42)	8,71 ns (2,29)	8,00 (2,93)	5,24 ns (2,51)
HADS-D	4,60 (2,99)	5,71* (2,75)	5,11 (2,99)	3,12 ns (3,33)

1. ns = not significant, * = < = 0.05, ** = < = 0.01, *** < = 0.001

All the secondary outcomes for girls in the intervention group improved, whereas for boys, the intervention did not result in the same improvement (Tables 3 and 4).

After controlling for initial values (WHO-5, RS, HADS-A, HADS-D, and SDQ), the intervention improved the OR of girls scoring higher on the follow-up WHO-5 (OR = 9.0; 1.4–56). The intervention did not improve the OR of higher scores in boys during follow-up (OR = 0.1; 0.09–1.4). This finding indicated that secondary school girls overall benefited somewhat more from this intervention, whereas boys did not to the same degree.

Feasibility studies are increasingly used to inform planning decisions for more definitive randomized controlled trials or other randomized designs. These studies can provide information on process measures, such as consent rates, treatment fidelity, and compliance, as well as on methods of outcome measurement. Additionally, they can provide initial parameter estimates for a sample size calculation, such as the standard deviation or the ‘success’ rate in the control group for a binary outcome. Between-group effects measured in feasibility studies are sometimes used to indicate the magnitude of an effect that might be obtained in a main trial, and a decision on progression is made with reference to the associated confidence interval. Such estimates will be imprecise in typically small studies. Within-group change might be estimated from a feasibility study to assess the potential efficacy of a novel intervention prior to testing it in a larger study, but, again, such estimates are liable to be imprecise and do not allow sound causal inferences.

## Students’ experiences

To deepen understanding of the quantitative results, students interviewed were about their experiences with the intervention, their overall attitudes towards the intervention, and the content and format of the intervention sessions. Focus group interviews revealed a positive overall attitude towards the intervention. To obtain an instant opinion from each participant, the students were asked to verbally rate the intervention on a scale from 1 to 10 (without further explanation). Their answers ranged from 4 to 9, with a type value of 7.

### Experienced values and overall opinions

In the interviews, all participating students were prompted to express their opinions and experiences. Still, the content of their answers varied greatly and the extent to which they were prepared to elaborate. Interviews lasted between 15 and 32 min, depending on how much the students participated and elaborated. To show the diversity and maximum variation in experiences, we present the extremes in citations from students who described their participation in the intervention.

*I thought it (the intervention) was perfect, and after each time, one felt more alert and a little happier, I think. In addition, I believe… in the long run, when we finished it all, it felt even better because you talked very openly with each other and got closer in the group. So that was fun (*Group 1, mixed sexes*).*

*There is so much fuzz with everything……What is wrong with just going to school, getting home when school ends, and trying to mind your own business? Instead of talking so dammed much about everything possible (*Group 3, only boys)

### SAW’s educational content

The students stated that the intervention content was familiar to them. However, it translated differently in the groups, suggesting a greater need for knowledge and discussion on various topics.

*I had no problem with it (the intervention), but I don't have to sit there and listen to things that I already know, for one and a half hours, when I have so much to study. It is just more stressful because I think about my other tasks (Group 2, mixed sex*).

*Everything we talked about was necessary; it was connected in a way to achieve good mental and physical health. Everything was needed in its own way, whether you have any use for it now or later in life (Group 1 mixed sexes*).

### SAW’s experimental content

The intervention also included an experimental part in each session, starting with an opening gesture in which a candle was lit and guided relaxation. In the middle of the session, the students took part in a creative exercise such as physical movement or playful games guided by the facilitators. This was regarded as the most controversial part of the intervention. Some students felt disturbed and openly questioned the creative exercise, whereas others saw the value of both relaxation and physical activities.

*We said no to that (lighting the candle). Well, we did (most of the experimental activities), but we are not directly preschoolers (Group 3 is* only boys*).*

*When we talked about chronic obstructive pulmonary disease, we had to do exercises with a balloon, jump, and so on. Games are fun because you need to be active … not just sit and chat … And when we changed chairs … that was also fun (Group 5,* only girls*).*

### SAW’s format

The session structure was identical, with the different parts following each other in a predetermined order. Not every student spoke out, but overall, students were positive or neutral. Some students were positive about the intervention, and the recurring structure was appreciated. Below is an example:

*It felt like you had this structure; we did things to make a certain impact —for example, the relaxation we had every time at the start. It was great when we sat there, just took it easy, and listened. You got very relaxed when you sat there, and similarly at the end when we all gathered and blew out the candle. Everyone knew what to do* (Group 4 mixed sexes).

### SHCTs’ experiences

Four focus group interviews were conducted with SHCT members to evaluate the intervention. The primary benefit of the intervention was the time spent with the students, which deepened the relationships between the individual SHCT member and the individual student, but also among the group of students. This made it possible to discuss different topics in a positive atmosphere. However, it wasn't easy to implement, as adjustments were necessary, which made the preparation time-consuming.

### Experienced values and overall opinions

Among the SHCTs, there were some doubts about the intervention's value and whether students would perceive it as meaningful and motivating. However, most students were involved in discussions and activities. The time spent with them was described as “a source of energy” where staff felt an energy recharge:

*It felt like you got a refill of force. You were on your toes and about to meet a group. However, then, yes, it gave energy (*SHTC group 3*).*

The importance of the intervention varied among team members. It meant an extra workload. Some were clear that they would not do it again under the same conditions, while others said explicitly that it was not at all burdensome. However, they emphasized increased contact with students, which strengthened their mutual relationships. It provided a new opportunity to work proactively within school health care:

*It was great fun; you get close to them... advantageously, we get to know the students better… It has started some processes in them, so it is … and these are the valves and shutters we need to open to do the preventive work* (SHCT group 1).

In all the SHCT interviews, the importance of the health dialogue was mentioned, as was the possibility of adjusting the content of the group sessions accordingly. The health dialogue between the school nurse and the student focuses on the individual student but also provides information about the school/class situation. In this way, SHCT can address the various problems and challenges across different classes.

### SAW’s educational content

There was a need to adapt the intervention's content to specific groups. Most students participated in the discussions, although not all topics generated the same level of interest. However, the SHCT team felt they could adapt and address students' comments. When social health (social capital, support, and structural factors) was the topic of the session, one student mentioned that a workplace-based learning period was about to begin. The session plan was changed, and the discussion shifted to “Who would you employ from an employer’s point of view?” The activity focused on social behaviour when meeting a potential employer, which was perceived as both fun and thought-provoking:

It is about to catch that little thing that arises (SHTC group 3).

Some of the SHCTs suggested that students would be involved in the design and, in that way, choose topics considered essential to discuss. Most members in the SHCTs were clear that preparation had to be done before the session:

*You cannot be unprepared, indeed not; you must have a presentation. On the other hand, to be compliant with the group, you must know what works and when it works* (SHCT group 3).

### SAW’s experimental content

The experimental content varied between the groups. Starting the session with a candlelight lightning round, followed by a short meditation, was not successful in all groups. Some students frankly declared that they would not participate in these sessions. The activities that worked well were described by other SHCTs as shown below:

*We had an exercise, like “the hot chair,” and talked about stress with music in the background: Mandala painting, condoms on wooden a penis, and many YouTube clips* (SHCT group 4).

### SAW’s format

The random composition of groups with six to ten students each worked well, and attendance was predominantly good. Initially, the atmosphere was slightly tense, but it gradually improved.

The SHCTs noted that as the sessions unfolded, the students became more engaged and spoke increasingly about personal matters. It was perceived as positive to participate in discussions without being evaluated or graded. Furthermore, the size of the group and the nondisclosure agreement (stating that all that was said in the room stayed in the room) created a safe and secure space:

*They (the students) have been very good at talking and discussing. You sense their need. Here, they have dared to ask questions to grasp what we were talking about. No, they truly were... engaged… Yes, great fun* (SHCT group 4).

## Discussion

According to the European Commission [14], schools across Europe need to promote and prioritize the mental health and well-being of schoolchildren through a safe, holistic approach to education that encompasses the social, emotional, and physical needs of children and adolescents. Adolescence is a crucial period laying the foundations for healthy development and mental well-being throughout life. School-based interventions to promote students’ mental health and well-being are more likely to be effective when organized within a systemic, whole-school approach than when implemented as single-component or fragmented interventions [39].

The SAW program is a means for such intervention, based on a conscious mix of core components, knowledge dissemination, stress management, reflective exercises, and creative activities, and adaptable components with lighting a candle and so on experimental parts (further, see [3]) aimed at supporting the whole person; the ability to be receptive, become socially involved and to verbalize and discuss topics relevant to the participants’ life situation.

In this study, there was a trend towards girls benefiting more from this intervention, as the intervention improved the WHO-5 OR scores of girls during follow-up (OR = 9.0; 1.4–56), but not for boys (OR = 0.1; 0.09–1.4). This finding may indicate that thesecondary school girls benefited from this intervention, whereas boys did not to the same degree. The qualitative findings on the girls are aligned with these results. Some of the somewhat unexpected results of the boys' different SDQ dimensions (emotional problems changing significantly from 2.2 to 4.9) and in, for instance, the HADS-D results (increasing significantly from 4.6 to 5.7), which contradict the interviews where the boys' group described that they had no need for the intervention. The qualitative findings also, to some extent, supported these gender differences. For example, interview group 3, consisting exclusively of boys, did not articulate any experienced or foreseeable benefit from the intervention, but rather questioned their presence in the intervention: “What is wrong with just doing schoolwork as usual?” On the other hand, the mixed group 1 and one of the groups consisting completely of girls (group 5), were very positive to both the educational content as well as to the adaptable components, indicating a more complex reaction to the intervention. This could be reflected by the intervention, which focused on discussions of abstract concepts. This may give girls a structural advantage over boys. With the current design, a certain level of verbal or social competence might be required to benefit from the present intervention [40]. Girls also tend to report more prosocial behaviours than boys do [41]. Prosocial behaviours include empathy, generosity, and helpfulness towards others, whereas difficulties include internalizing problems and externalizing problems, such as acting out. Students’ perceptions of the intervention varied but were generally positive, which is essential, as the reporting of multiple self-reported health complaints has more than doubled since the mid-1980s [42].

Some parts of the SAW format were considered controversial by some students, leading some groups to change the format to exclude them. While lighting a candle and some creative content might not be crucial to the intervention's overall effect, they could indicate a willingness to participate in the SAW program. Such hostility could mean many things, but if it led students to shun health promotion, it may have led to a lack of effect. Hopefully, SHCTs' ability to adapt to students in their groups could lessen this resistance and increase students’ susceptibility to SHCT health promotion efforts. To some extent, this is reflected in the SHCT interviews.

Our preliminary findings, based on a small, non-randomised pilot, indicate that intervention is one way to strengthen adolescents’ health. The content of interventions, such as mental health, physical activity, and sleeping habits, is related to school performance. A recent study revealed an association between self-reported health and school experience among high school students (Forsberg et al. [43]).

The SHCTs appreciated the time spent with the students, as it brought them closer together, and the content and form were well-received. However, being a facilitator was challenging. The sessions had to be planned, and no additional resources were allocated from the school management. It was a new way of working for most members of the SHCT, who felt both demanding and exciting. It was also implemented during a period of extensive reorganization in the municipality's schools, which delayed its implementation. This caused uncertainty about when to start, and which classes and teams would be involved. Issues that could have indirectly affected the experience of implementing the intervention. The SHCTs clearly revealed that the SAW intervention needed to be adapted to each group. They all mentioned health dialogue as a valuable means of promoting adolescents’ health. The possibility of gaining insights into both the individual student’s and the class's situations could enable the SAW intervention to be adapted as effectively as possible. The health dialogue was developed by the school health service with the ambition of promoting students’ health [18].

## Methodological considerations

The main school director determined the group allocation, deciding which of the two possible schools would serve as the control school. We are aware that this is a potential source of selection bias, as, for instance, there could be differences not accounted for in academic admission scores. Random allocation of students to conditions was not possible, as intervention schools were required to allocate time for group intervention, have at least one SHCT member who had received SAW training, and have the capacity to implement the intervention during a prespecified intervention period. Such constraints are not uncommon in school-based research. Furthermore, a sample size calculation was needed to detect meaningful differences in WHO-5 scores, based on previous applications of the questionnaire. The final sample size was 98, suggesting the study was marginally underpowered. Finally, although the schools included in this study may represent children from across the socioeconomic spectrum, differences between intervention and control school characteristics warrant further exploration. This is particularly important for improving the understanding of the impact of school-based interventions on reducing health inequalities.

The intermediate group was defined as those individuals most likely to be affected by the intervention. A lower WHO-5 score, i.e., below or equal to 20, may be indicative of mental illness [26]. When analysing the sample, the outliers were excluded, and only answers between the 12.5th percentile and the 87.5th percentile were used for further analysis. In addition, this corresponded to the WHO-5 three-middle group (21–80) total score, excluding those who were below 20 or above 80 at the pretest (baseline).

To address baseline imbalances and the small study size, a future implementation of a modified SAW intervention would use a cluster-randomised design, with municipalities defining the clusters. Secondly, it would, as in the current study, include all school classes at the addressed grade (age-cohort) at the individual school and all students in those classes.

## Conclusion

We conclude that the design of the SAW intervention is a feasible health promotion in the setting of Swedish Upper Secondary schools. The intervention was considered acceptable among staff and girls. Though the intervention may need some modifications to accommodate the boys' experience before further implementation. Additionally, the SAW intervention requires adequate resources at the school level to be fully implemented. The differences between the experimental and control groups' outcomes were minor; however, we casually conclude that the girls may benefit more from this intervention than the boys did. This could be the most valuable result of the intervention. For this age group, it is therefore important to design the future SAW interventions on mental well-being where gender is explicitly taken into consideration, with different programs respectively directed to boys and girls. In contrast to time-consuming aspects, the SHCTs appreciated the intervention, as it provided a helpful tool and model for conducting school-based, interprofessional health-promoting work in line with school law. But these promising effects need to be confirmed in a larger, well-controlled study. SAW intervention would use a cluster randomisation design with different municipalities defining the cluster level, as it also provides us with a larger sample to generate more powered and reliable data, and therefore conclusions.

## Data Availability

It is not possible to take part in the data.
